# Molecular Dynamics Analysis of Graphene Nanoelectromechanical Resonators Based on Vacancy Defects

**DOI:** 10.3390/nano12101722

**Published:** 2022-05-18

**Authors:** Wenhua Li, Wenchao Tian

**Affiliations:** School of Electro-Mechanical Engineering, Xidian University, Xi’an 710071, China; wenhuali@stu.xidian.edu.cn

**Keywords:** graphene, defects, resonant

## Abstract

Due to the limitation of graphene processing technology, the prepared graphene inevitably contains various defects. The defects will have a particular influence on the macroscopic characteristics of the graphene. In this paper, the defect-based graphene nanoresonators are studied. In this study, the resonant properties of graphene were investigated via molecular dynamic simulations. The effect of vacancy defects and hole defects at different positions, numbers, and concentrations on the resonance frequency of graphene nanoribbons was studied. The results indicated that single monatomic vacancy has no effect on graphene resonant frequency, and the concentration of the resonant frequency of graphene decreases almost linearly with the increase of single-atom vacancy concentration. When the vacancy concentration is 5%, the resonance frequency is reduced by 12.77% compared to the perfect graphene. Holes on the graphene cause the resonance frequency to decrease. As the circular hole defect is closer to the center of the graphene nanoribbon, not only does its resonant frequency increase, but the tuning range is also expanded accordingly. Under the external force of 10.715 nN, the resonant frequency of graphene reaches 429.57 GHz when the circular hole is located at the center of the graphene nanoribbon, which is 40 GHz lower than that of single vacancy defect graphene. When the circular hole is close to the fixed end of graphene, the resonant frequency is 379.62 GHz, which is 90 GHz lower than that of single vacancy graphene. When the hole defect is at the center of nanoribbon, the frequency tunable range of graphene reaches 120 GHz. The tunable frequency range of graphene is 100.12 GHz when the hole defect is near the fixed ends of the graphene nanoribbon. This work is of great significance for design and performance optimization of graphene-based nanoelectro-mechanical system (NEMS) resonators.

## 1. Introduction

As a novel two-dimensional nanomaterial composed of sp^2^ hybrid carbon atoms [1], graphene has excellent properties, such as superior thermal conductivity, high electronic conductivity, and outstanding mechanical properties [2,3,4,5]. Therefore, graphene is considered to be an ideal material for nanoelectro-mechanical system (NEMS) resonators [6]. Graphene-based nanomechanical resonators have attracted a lot of attention [7]. It has high frequency, strong mechanical nonlinearities, high impact factor, small volume and mass, and ultra-high sensitivity [8]. Graphene-based nanomechanical resonators have a broad prospect in many fields, such as radio frequency (RF) communication [9], microwave devices [10], high sensitivity sensors [11], single molecule detectors [12], and macroscopic quantum effect detection [13]. Bunch et al. first fabricated a graphene mechanical resonator with the length and width in micrometer size. The fundamental frequency is on the order of MHz [14]. Jiang et al. studied the free vibration of single-layered graphene-based mass sensor by Galerkin strip DFT method [15]. Numerous experimental and theoretical studies have thus been carried out to utilize graphene in nanomechanical resonators.

**Figure 1:**
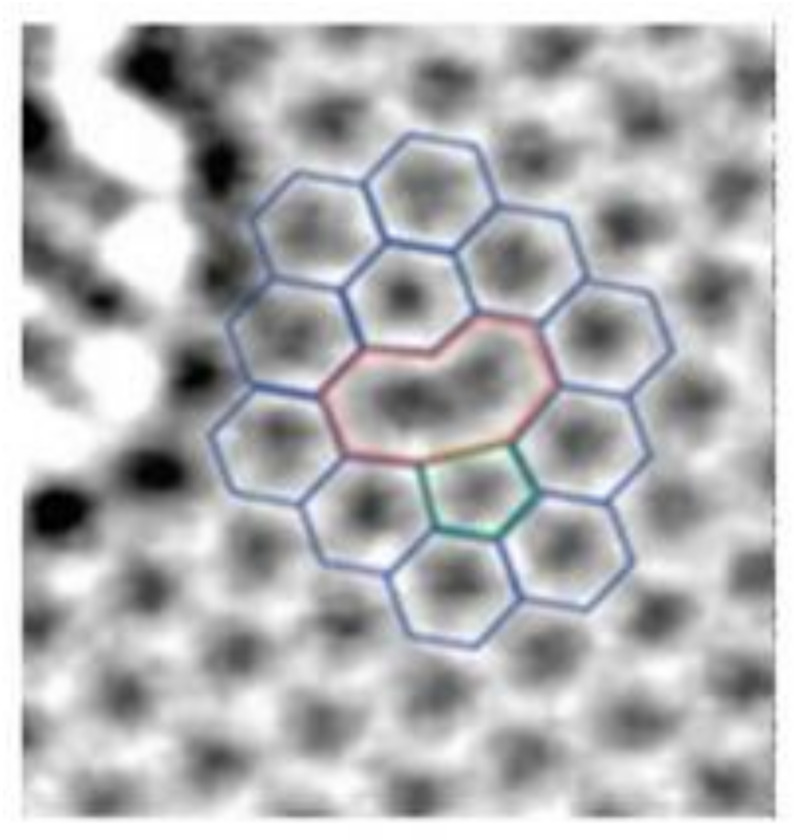
Graphene single hole defect (reprinted with permission from [21]. Copyright 2012 American Chemical Society).

However, graphene is not perfect during the production process and structural defects are inevitable [16]. Graphene defects can be divided into three types according to how the defects are created: thermally dynamically resultant, deformation-introduced, and artificially-induced defects [17]. The formation energy of thermally activated defects are generally less, such as point vacancy defects and Stone–Wales defects (SW) [18]. Chemical vapor deposition (CVD) method enables mass production of large-area graphene. However, graphene fabricated by CVD method and epitaxial growth unavoidably contain line defects, such as gain boundary [19]. Adatom defects can be obtained by various chemical and heat treatments [20]. Defects also can be artificially introduced by electron and ion beam irradiation, such as nanopores and holes. Furthermore, the size of nanopores can be controlled by focused ion beam (FIB). Deletion of a carbon atom in the six-membered ring lattice of graphene leads to a single-atom vacancy defect. Figure 1 shows the transmission electron microscope image and atomic structure diagram of a single vacancy defect [21].

The presence of defects breaks the symmetric lattice structure of graphene, which has a significant impact on the mechanical, thermal, and electrical properties. Due to this reason, a lot of experimental and theoretical research has concentrated on investigating the effects of these defects. For instance, Li et al. found that the mechanical properties were significantly reduced with the existence of defect by the molecular dynamic (MD) simulations, and the thermal conductivity of graphene containing SV, DV, and SW decreased ~57.6%, ~43.4%, and ~31% [22]. Ahangari et al. indicated that the point (Stone–Wales (SW) and atom vacancies) and shape defects had a negative effect on Young’s modulus using ab-initio-based density functional theory (DFT) calculations [23].

In the literature, abundant experimental and theoretical simulation reports are available on the effects of various defects on the thermal conductivity [24], electron density [25], and mechanical strength [26,27,28] of pristine graphene. The effects of the type, location, and concentration of defects on the resonant properties of graphene have been rarely reported, and the behavior of defective graphene NEMS resonators is not well understood. Thus, it is of great significance to investigate the resonate properties of graphene and effects of various defects. As the device develops towards miniaturization, the mechanical model based on the traditional macroscopic continuum is no longer applicable when the scale enters the nanometer level. Molecular dynamics simulation can be used to study the configuration and energy changes of graphene materials at resonance with atomic accuracy [29]. In this paper, we have conducted a series of MD simulations to explore the resonance law of defective graphene. The effect of vacancy defects and hole defects at different positions, numbers, and concentrations on the resonance frequency of graphene nanoribbons is studied via MD simulation. The results provide necessary theoretical guidance for the design and performance optimization of graphene-based NEMS resonators. The findings can provide a comprehensive understanding in the resonante properties of defective graphene.

## 2. Materials and Methods

**Figure 2:**
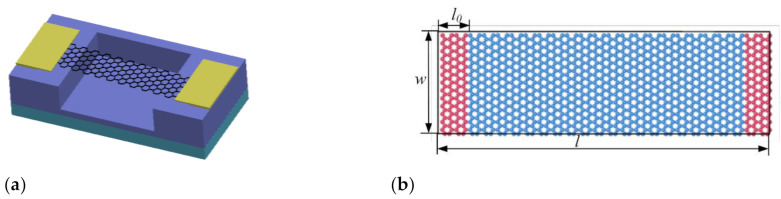
Schematic diagram of graphene nanoribbon model, (**a**) Schematic diagram of double-end solid-supported graphene resonator structure, (**b**) Schematic diagram of graphene resonant beam.

Molecular dynamics simulation is one of the effective methods to study the characteristics of micro-nano structures [30]. It deeply describes the complex mechanical mechanism of discrete atomic and molecular systems, shows the characteristics that cannot be achieved by experiments and continuum theory, and can accurately carry out numerical calculation. The molecular models were first constructed. The double-ended fixed-branch graphene nanoribbon established in this simulation is shown in Figure 2, which is armchair-shaped in the length direction. The length l is 10.224 nm and the width w is 3.19736 nm. The molecular model of graphene nanoribbon consisted of 1248 carbon atoms. During the actual experiment, the graphene has to be placed on a SiO_2_ substrate, therefore the two ends of the graphene nanoribbon structure are fixed in the simulation model. The red area at both ends is the fixed part, and the fixed atomic number is 104 × 2. The middle blue region is the vibrating part, which contains 1020 carbon atoms. The distance from the graphene resonant beam to the substrate is 5 nm.

In this paper, Large-scale Atomic/Molecular Parallel Simulation (LAMMPS) software (Version 24Dec2020, Sandia National Laboratories, Albuquerque, NM, USA) is used for simulation. The time step is 1 × 10^−3^ ps (1 fs). The Boltzmann constant in the system is 1.38 × 10^−23^ J/K. An Adaptive Intermolecular Reactive Empirical Bond Order (AIREBO) potential function is used to simulate the C–C interaction. The AIREBO potential function includes not only the interaction force generated by the potential but also the long-range interaction force. The C-C bond length is 3.4 Å. The cut-off radius is set to 10.2 Å. In the system numerical solution uses the Velocity-Verlet algorithm with high computational efficiency. The boundary condition of setting the nanoribbon X direction in the horizontal plane is periodic boundary condition. The boundary condition of the nanoribbon in the Y direction is periodic boundary condition. An infinite parallel plate capacitor is simulated. A boundary condition is set as a fixed boundary condition in the normal direction of the capacitor, that is, in the Z direction. Nosé-Hoover thermostat was used to control the temperature in the simulation.

The external force applied in this paper is calculated based on the electrostatic force of an infinite parallel plate capacitor. The electrostatic force between the substrate and the resonant beam can be calculated by the following formula without considering the edge effect [31]:

(1) $$F\;=\;-\frac{\varepsilon \;WL}{2(d_{0}-w)}V^{2}\cdot F\;=\;-\frac{\varepsilon \;WL}{2(d_{0}-w)}V^{2}$$

Among them, *W* is the potential energy of the resonator, *ε* is the dielectric constant, *L* is the length of the resonant beam, *w* is the deflection of the resonant beam, *V* is the voltage between the upper and lower plates, including direct current (DC) bias voltage and small alternating voltage. Derivation of potential energy in displacement direction is the magnitude of electrostatic force between two plates:

(2) $$F\;=\;\frac{\partial W}{\partial d_{0}}\;=\;\frac{1}{2}\frac{\partial C}{\partial d_{0}}U^{2}\;=\;-\frac{\varepsilon abU^{2}}{2d_{0}{}^{2}}$$

The minus sign represents the direction of electrostatic attraction between the parallel plates of the capacitor. The external force in molecular dynamics simulation is the electrostatic force calculated by Formula (2), which is uniformly distributed on each atom in the effective region of graphene nanoribbons.

## 3. Results and Discussion

### 3.1. Effect of Single Void Defects on the Resonance Frequency of Graphene

**Figure 3:**
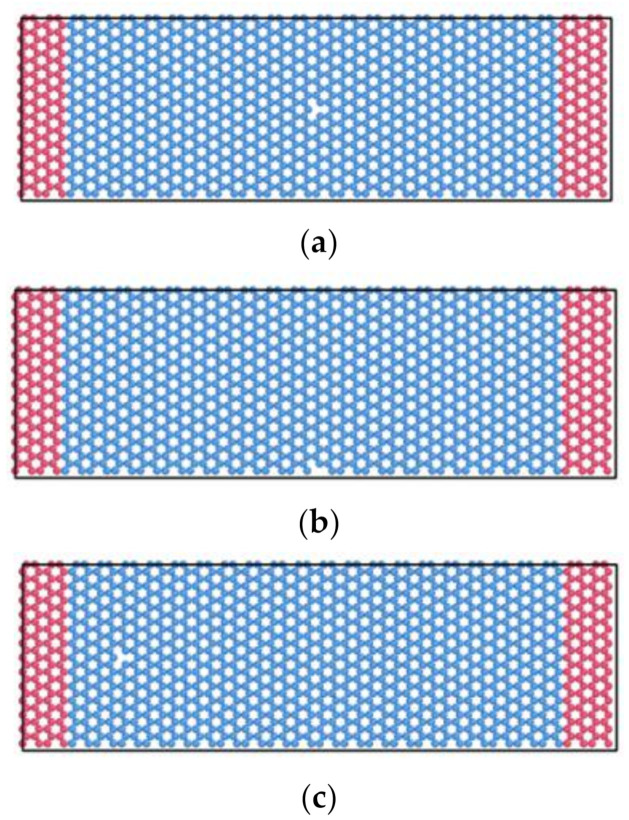
Schematic diagram of three type graphene nanoribbons containing single atom vacancy. (**a**) SV-1 model of the single atom vacancy at the center position. (**b**) SV-2 model of the single atom vacancy at the edge position. (**c**) SV-3 model of the single atom vacancy at other locations.

Considering the effects of the location, size, and concentration of vacancy defects on the resonant properties of graphene, single-atom vacancies were set on the graphene nanoribbon. Atoms D1 (51.119, 14.757, 0) at the center position, D2 (51.119, 0, 0) at the edge position, and D3 (17.040, 14.757, 0) at the other positions were removed from the model, respectively. The corresponding structures of graphene nanoribbon models SV-1, SV-2, and SV3 containing single vacancies were obtained, as shown in Figure 3.

**Figure 4:**
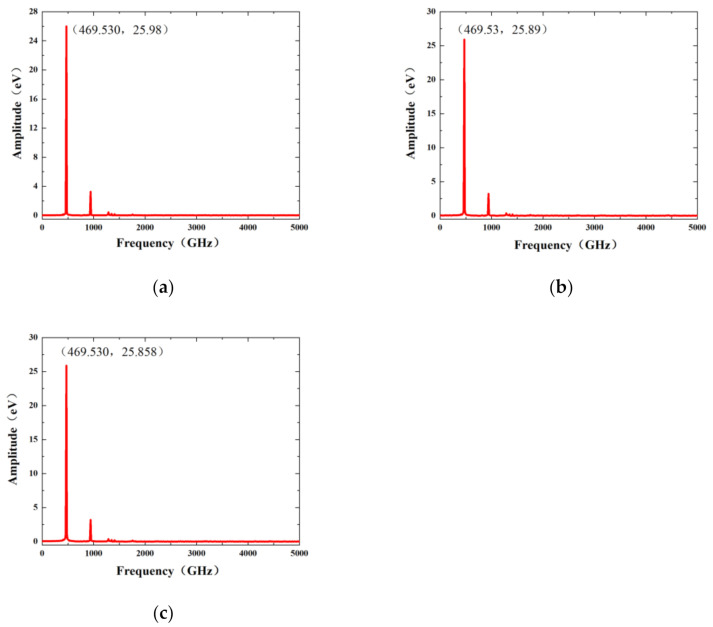
Frequency response curve of single-atom vacancy graphene under 10.175 nN external force: (**a**) SV-1 frequency response curve, (**b**) SV-2 frequency response curve, (**c**) SV-3 frequency response curve.

In the molecular dynamics simulation, the potential function selects the AIREBO potential. The time step is set to 1 fs, and relaxation of 50,000 time steps is performed first. After the relaxation was completed, external forces of 0.297 nN, 1.191 nN, 4.762 nN, and 10.715 nN were loaded, respectively. After that, the energy was minimized again. Then the external force was removed, and the NVT ensemble was changed to the NVE ensemble. At this moment, the kinetic energy of the system and the potential energy between the bonding atoms transform to each other, and graphene nanoribbons vibrate freely. Selecting the kinetic energy or potential energy change of the system as the time-domain signal, the corresponding frequency response curve can be obtained by Fourier transform. The graphene resonant frequency of the different vacancy positions under the action of 10.715 nN external force is shown in Figure 4.

**Figure 5:**
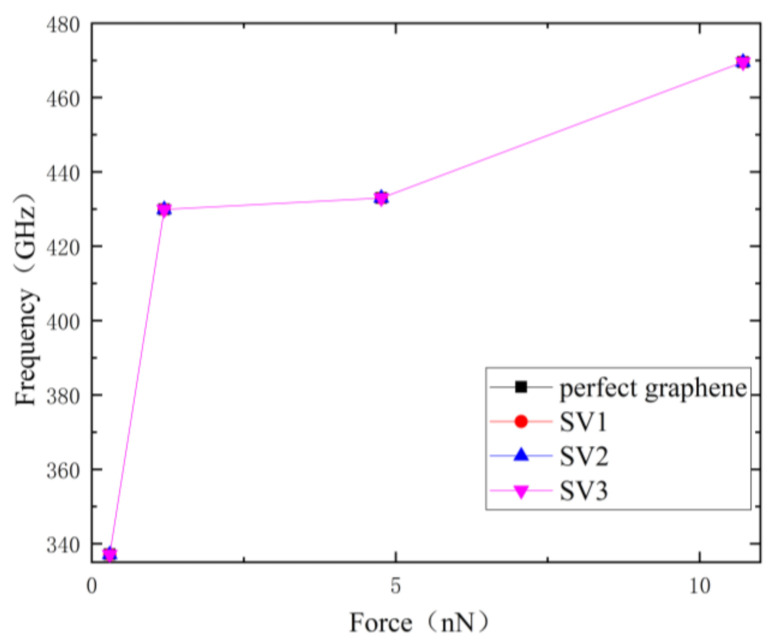
Resonant frequency variation curve of three graphene nanoribbons with different external force.

As can be seen in Figure 5, the first-order resonant frequencies of single-atom vacancy nanoribbons at different locations basically overlap. Point M in the figure indicates that the resonant frequency magnitude of graphene is 337.12 GHz under the external force of 0.297 nN, point N indicates that its resonant frequency is 429.9 GHz under the external force of 1.191 nN, point P indicates that the resonant frequency slowly increases to 433.65 GHz when the external force continues to increase to 4.762 nN. Point Q indicates that when the external force is 10.715 nN, the single-vacancy defect graphene at three positions and perfect graphene shows the same resonant frequency, which is 469.53 GHz. The increase in resonant frequency with different applied stresses is consistent with that of the perfect graphene. The resonant frequency of the graphene structure shows an increasing trend with increasing loading forces, which is nonlinear. For the case of consistent frequencies of single-atom vacancy graphene in different arrangements, probably because the loss of a single atom has little effect on the whole graphene nanoribbon system. There is not enough precision to distinguish the minor differences in the statistical physics used in this paper. The loss of a single atom does not change the resonant properties of the graphene nanoribbon in molecular dynamics simulations.

**Figure 6:**
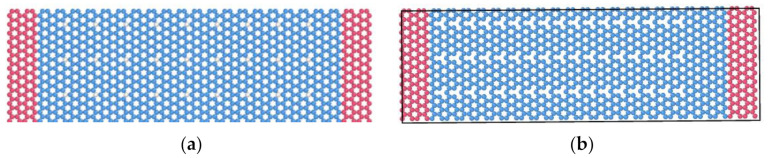
Schematic diagram of the graphene nanoribbon model with different concentrations. (**a**) Graphene nanoribbons with a vacancy concentration of 2.647%. (**b**) Graphene nanoribbons with a vacancy concentration of 5%.

**Figure 7:**
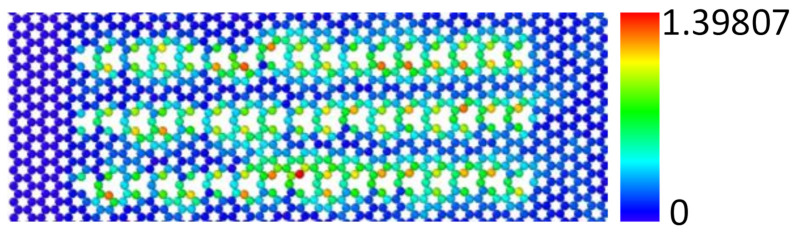
Displacement cloud of graphene nanoribbon with vacancy concentration of 5% at a relaxation time of 10 ps.

Since the effect of a single atomic vacancy on the resonant frequency of graphene nanoribbons is small, the number of defects is increased to consider the impact of different defect concentrations on the resonant frequency of graphene. In fact, vacancy defects are the most typical defects that can exist stably in graphene and will inevitably be generated in the processing of graphene. For example, when graphene is treated by ion irradiation technology, the number of defects increase with the increase of irradiation dose, and the defects are mainly vacancies. In this paper, the concentration of vacancy defects is expressed as the ratio of missing carbon atoms to the total number of intact graphene atoms. The graphene nanoribbons with vacancy defect rates of 0.294%, 1.4705%, 2.647%, 3.8235%, and 5% were modelled, and the distribution of vacancies on the graphene nanoribbons remained symmetrical and uniform. Figure 6 shows the structure of the vacancy graphene model with different concentrations. Firstly, the graphene nanoribbon model was subjected to a 50,000-step relaxation. The atomic displacement deformation clouds chart during the relaxation process are shown in Figure 7. It can be seen that the carbon atoms near the single-atom vacancy have a larger displacement than carbon atoms in other positions. This is because the carbon atoms near the vacancy have lost a covalent bond between carbon and carbon atoms. Graphene nanoribbons have dangling bonds and are structurally unstable, so these atoms fluctuate more during relaxation.

**Figure 8:**
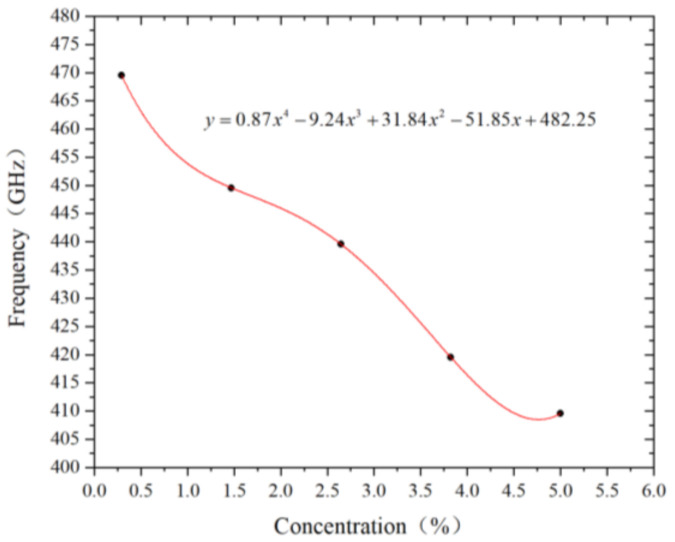
Graphene resonant frequency variation polynomial fitting curve with vacancy concentration.

Figure 8 shows the effect of different vacancy concentrations on the resonant frequency of graphene. It can be seen that the resonant frequency of graphene nanoribbons decreases almost linearly with the increase of vacancy defect concentration. The vibration amplitude of resonant bands also shows a general decreasing trend. When the concentration of vacancy defects is 0.294%, the number of single-atom vacancy is 3. It is the same as the resonant frequency of graphene with one single-atom vacancy defect. When the concentration increases to 5%, the resonant frequency of graphene decreases by 12.77%. In contrast, the concentration of defects in the graphene nanoribbon significantly affects its resonant frequency.

In the Euler–Bernoulli beam theory [14], the resonant frequency of the double-ended solidly supported beam when the stress σ acts uniformly on it is given by

(3) $$\begin{array}{l} f\;=\;{\left\{{\left[\frac{At}{t^{2}}\sqrt{\frac{E}{\rho }}\right]}^{2}+\frac{0.57A^{2}\sigma }{\rho l^{2}wt}\right\}}^{1/2}\\ \;=\;{\left\{{\left[\frac{At}{t^{2}}\sqrt{\frac{E}{\rho }}\right]}^{2}+\frac{0.57A^{2}\left(E\varepsilon_{1}+D\varepsilon_{1}^{2}\right)}{\rho l^{2}wt}\right\}}^{1/2} \end{array}$$

where *E*, *D*, *ρ*, *l*, *w*, and *t* are Young’s modulus, third-order modulus of elasticity, density, length, width, and beam thickness, respectively. Obviously, from Equation (3), the fundamental frequency of graphene nanoribbons decreases with decreasing elastic modulus without changing the size of the model and without strain. The simulation results of Han et al. showed that single atomic and diatomic vacancy defects have essentially no effect on the elastic modulus of graphene films [32]. Therefore, in the simulation of graphene nanoribbons containing only one vacancy defect, the reason why different vacancy positions did not change the resonant frequency may be because a single-atom vacancy did not change the Young’s modulus of graphene. For graphene models with different defect concentrations, Zandiatashbar et al. investigated the effect of defects on the mechanical properties of graphene. They showed that the elastic modulus did not change even with a high concentration of hybridized carbon atoms, while vacancy defects significantly reduced the resonant frequency of grapheme [33]. According to Equation (3), the resonance frequency of the nanoribbon is proportional to the Young’s modulus. Therefore, when the number of defects increases, that is, the concentration increases, the decrease in the resonant frequency may be caused by the decrease in Young’s modulus.

### 3.2. Effect of Circular Hole Defects on the Resonant Frequency of Graphene

**Figure 9:**
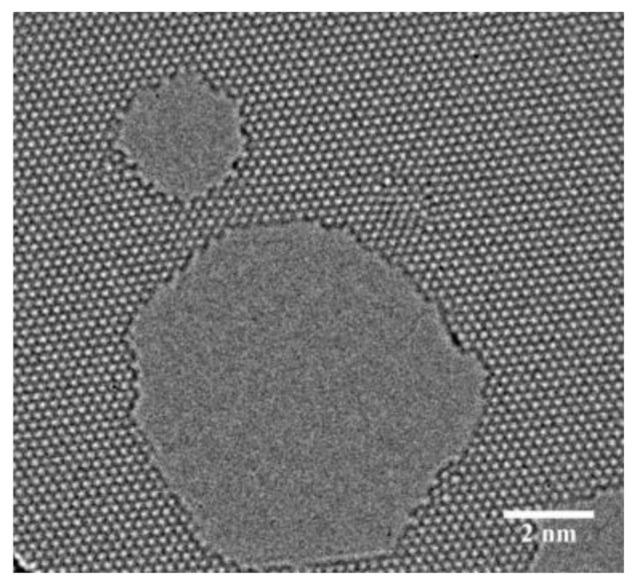
TEM image of small holes on monolayer graphene produced by electron beam sputtering (reprinted with permission from [34]. Copyright 2012 American Chemical Society).

Circular hole defects are also one of the common defects in graphene. For example, in the preparation process of reduced graphene oxide there is a strong oxidation reaction, which leads to the loss of atoms and causes defects such as holes. Continuous electron beam irradiation will also cause small holes in the graphene film. Figure 9 shows the spherical aberration correction transmission electron microscope (TEM) image of monolayer graphene with small holes [34].

**Figure 10:**
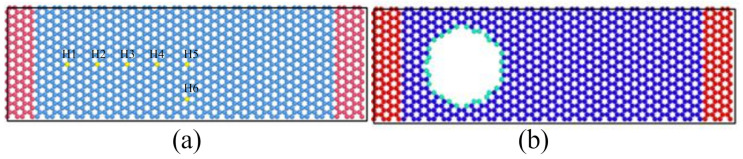
(**a**) Schematic diagram of the location of the circle center of the circular hole defect on graphene, (**b**) Graphene model of the circular hole defect with circle center location H3.

Fabricate hole defects on perfect graphene nanoribbons: removal of all atoms in the circle with atomic H as the center and r = 0.1 nm as the radius of the hole. The positions of the centers of the circles are H_1_ (17.040, 14.757), H_2_ (25.559, 14.757), H_3_ (34.080, 14.757), H_4_ (42.599, 14.757), H_5_ (51.119, 14.757), H_6_ (51.119, 6.595) on the graphene nanoribbons are shown in Figure 10a, where the yellow atoms indicate the circular hole centers. The model graphene nanoribbons containing circular hole defects are numbered as H1, H2, H3, H4, H5, and H6.

**Figure 11:**
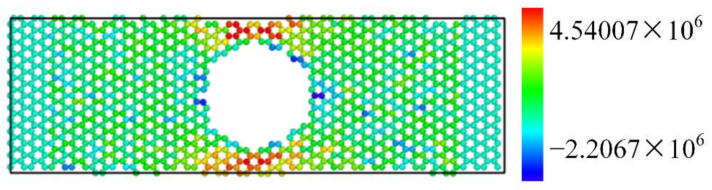
Graphene stress cloud chart during relaxation.

The parameters used are the same as those used for the simulation of single-atom defects. First, the relaxation process is analyzed, and the two ends of the graphene structure are fixed. Then, the full relaxation is carried out to minimize the energy of the system. The stress cloud chart in the relaxation process is shown in Figure 11. It can be seen that the atoms around the hole are less stressed. This is because there are only a few C-C bonds around these carbon atoms, which are bound by small interatomic forces. The stress near the edge of the nanoribbon is relatively large, which may have a certain impact on the resonant frequency of graphene.

**Figure 12:**
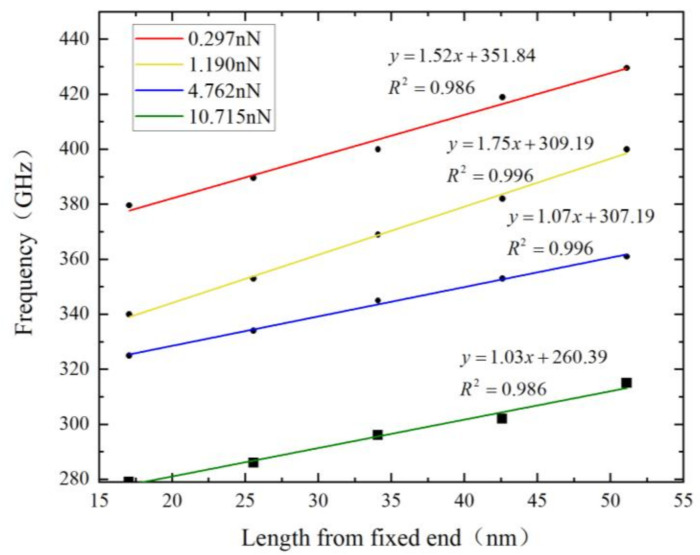
Variation of graphene resonant frequency with circular hole defect location under different external forces.

When sufficient relaxation is carried out, external forces of 0.297 nN, 1.191 nN, 4.762 nN, and 10.715 nN are loaded. After the stress is loaded, the original NVT ensemble is transformed into an NVE ensemble. Then the applied load is removed, and graphene enters the free vibration phase. The kinetic energy of the system is used as the time domain signal for the analysis of resonant frequency calculation. The resonant frequencies of the circular hole nanoribbons at different locations under each external force are statistically analyzed. The relationship between the resonant frequencies of graphene and different external loads, as well as different circular hole defect locations, is shown in Figure 12. It can be seen that the closer the circular hole position is to the graphene center, the resonant frequency of graphene increases. This increase trend is almost linear. Under the external force of 10.715 nN, the resonant frequency of graphene reaches 429.57 GHz when the circular hole is located at the center of the graphene nanoribbon, i.e., H5 position, which is 40 GHz lower than that of single vacancy defect graphene. When the circular hole is close to the fixed end of graphene, that is, the H1 position, the resonant frequency is 379.62 GHz, which is 90 GHz lower than that of perfect graphene. The change trend of the resonance frequency with the position of the circular hole is consistent under different external force loads.

**Figure 13:**
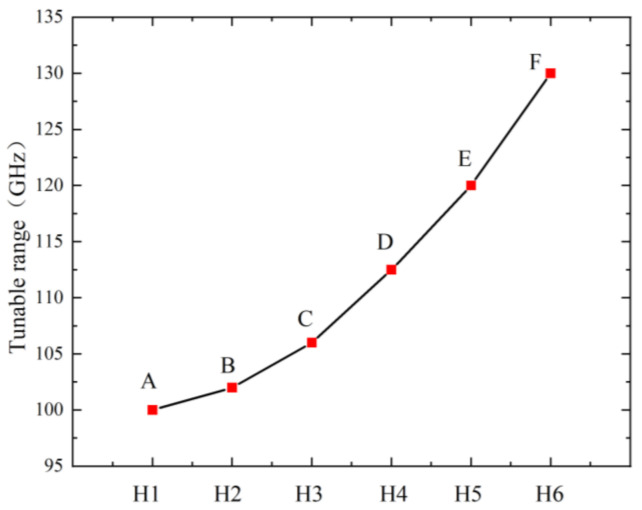
Graph of the variation of graphene tunable range with the position of the center of the hole defect.

Figure 13 shows the effect of the location of the circular hole defect on the resonant frequency of graphene. It can be seen that as the circular hole gets closer to the center of the graphene nanoribbon, the tunable range of graphene gradually increases, and the rate of increase becomes faster and faster. That is, the variation range of resonant frequency caused by different external forces is larger. For electrostatically driven resonators, a large tuning range can achieve higher sensitivity. Point E indicates that when the center of the hole defect is at the center of the nanobelt, that is, the H5 position, the frequency tunable range of graphene reaches 120 GHz. The tunable frequency range of graphene is 100.12 GHz when the center of the circular hole defect is near the fixed ends of the graphene nanoribbon. As the circular hole defect is closer to the center of the graphene nanoribbon, not only does its resonant frequency increase, but the tuning range is also expanded accordingly. Therefore, the circular hole defect should be located in the center of the graphene nanoribbon.

## 4. Conclusions

Studying the effect of defects on nanomechanical resonators based on graphene is essential for obtaining a high-quality graphene resonator. In this paper, we employed molecular dynamic method to investigate the effects of vacancy defects and hole defects on vibration characteristics of graphene-based nanomechanical resonator. First, the vacancy defects of different positions and concentrations are modeled in detail. The LAMMPS software was used to simulate the resonance characteristics of the models. The relaxation performance and the frequency response characteristics of graphene with vacancies under different external loads were emphatically analyzed. The results show that the resonant frequency of graphene nanoribbon increases in a step-like manner as the external force increases. The non-linear deformation dominates the dynamic behavior of graphene. By Fourier transform calculation, we found that single monatomic vacancy has no effect on graphene resonant frequency, and the concentration of the resonant frequency of graphene decreases almost linearly with the increase of single-atom vacancy concentration. In the next stage, nanohole defects were imposed to the graphene nanoribbon. The effect of hole location on the resonant frequency of graphene was analyzed and the simulation results show that holes obviously reduce the resonant frequency of graphene. As the circular hole defect is closer to the center of the graphene nanoribbon, not only does its resonant frequency increase, but the tuning range is also expanded accordingly. The above findings can provide a comprehensive understanding in the resonant properties of defective graphene. Our work provides a necessary theoretical foundation for the following research on the design and performance optimization of graphene nanoresonators.

## Data Availability

The data presented in this study are available on request from the corresponding author.

## References

[1] Das S., Robinson J.A., Dubey M., Terrones H., Terrones M. (2015). Beyond graphene: Progress in novel two-dimensional materials and van der Waals solids. Annu. Rev. Mater. Res..

[2] Ye R., Tour J.M. (2019). Graphene at fifteen. ACS Nano.

[3] Yu W., Sisi L., Haiyan Y., Jie L. (2020). Progress in the functional modification of graphene/graphene oxide: A review. RSC Adv..

[4] Balandin A.A. (2020). Phononics of graphene and related materials. ACS Nano.

[5] Lin L., Peng H., Liu Z. (2019). Synthesis challenges for graphene industry. Nat. Mater..

[6] Lee I.-H., Yoo D., Avouris P., Low T., Oh S.-H. (2019). Graphene acoustic plasmon resonator for ultrasensitive infrared spectroscopy. Nat. Nanotechnol..

[7] Nickpay M.-R., Danaie M., Shahzadi A. (2021). Highly sensitive THz refractive index sensor based on folded split-ring metamaterial graphene resonators. Plasmonics.

[8] Tiwari S.K., Sahoo S., Wang N., Huczko A. (2020). Graphene research and their outputs: Status and prospect. J. Sci. Adv. Mater. Devices.

[9] Awan S., Lombardo A., Colli A., Privitera G., Kulmala T., Kivioja J., Koshino M., Ferrari A. (2016). Transport conductivity of graphene at RF and microwave frequencies. 2D Mater..

[10] Meng F., Wang H., Huang F., Guo Y., Wang Z., Hui D., Zhou Z. (2018). Graphene-based microwave absorbing composites: A review and prospective. Compos. Part B Eng..

[11] Choi J.H., Lee J., Byeon M., Hong T.E., Park H., Lee C.Y. (2020). Graphene-based gas sensors with high sensitivity and minimal sensor-to-sensor variation. ACS Appl. Nano Mater..

[12] Kovalska E., Lesongeur P., Hogan B., Baldycheva A. (2019). Multi-layer graphene as a selective detector for future lung cancer biosensing platforms. Nanoscale.

[13] Kurniawan D., Jhang R.-C., Ostrikov K.K., Chiang W.-H. (2021). Microplasma-Tunable Graphene Quantum Dots for Ultrasensitive and Selective Detection of Cancer and Neurotransmitter Biomarkers. ACS Appl. Mater. Interfaces.

[14] Bunch J.S., van der Zande A.M., Verbridge S.S., Frank I.W., Tanenbaum D.M., Parpia J.M., Craighead H.G., McEuen P.L. (2007). Electromechanical resonators from graphene sheets. Science.

[15] Jiang R.W., Shen Z.B., Tang G.J. (2016). Vibration analysis of a single-layered graphene sheet-based mass sensor using the Galerkin strip distributed transfer function method. Acta Mech..

[16] Chu K., Wang J., Liu Y.-P., Geng Z.-R. (2018). Graphene defect engineering for optimizing the interface and mechanical properties of graphene/copper composites. Carbon.

[17] Sun X., Huang C., Wang L., Liang L., Cheng Y., Fei W., Li Y. (2021). Recent progress in graphene/polymer nanocomposites. Adv. Mater..

[18] Kotakoski J., Mangler C., Meyer J.C. (2014). Imaging atomic-level random walk of a point defect in graphene. Nat. Commun..

[19] Liu T.-H., Gajewski G., Pao C.-W., Chang C.-C. (2011). Structure, energy, and structural transformations of graphene grain boundaries from atomistic simulations. Carbon.

[20] Patera L.L., Bianchini F., Africh C., Dri C., Soldano G., Mariscal M.M., Peressi M., Comelli G. (2018). Real-time imaging of adatom-promoted graphene growth on nickel. Science.

[21] Meyer J.C., Kisielowski C., Erni R., Rossell M.D., Crommie M., Zettl A.J. (2008). Direct imaging of lattice atoms and topological defects in graphene membranes. Nano Lett..

[22] Li M., Zhou H., Zhang Y., Liao Y., Zhou H. (2018). Effect of defects on thermal conductivity of graphene/epoxy nanocomposites. Carbon.

[23] Ahangari M.G., Mashhadzadeh A.H., Fathalian M., Dadrasi A., Rostamiyan Y., Mallahi A. (2019). Effect of various defects on mechanical and electronic properties of zinc-oxide graphene-like structure: A DFT study. Vacuum.

[24] De Silva K.K.H., Huang H.-H., Joshi R., Yoshimura M. (2020). Restoration of the graphitic structure by defect repair during the thermal reduction of graphene oxide. Carbon.

[25] Singla M., Jaggi N. (2021). Enhanced hydrogen sensing properties in copper decorated nitrogen doped defective graphene nanoribbons: DFT study. Phys. E Low-Dimens. Syst. Nanostruct..

[26] Gupta K., Mukhopadhyay T., Roy A., Roy L., Dey S. (2021). Sparse machine learning assisted deep computational insights on the mechanical properties of graphene with intrinsic defects and doping. J. Phys. Chem. Solids.

[27] Lopez-Polin G., Gomez-Navarro C., Gomez-Herrero J. (2022). The effect of rippling on the mechanical properties of graphene. Nano Mater. Sci..

[28] Huang P., Li Y., Yang G., Li Z.-X., Li Y.-Q., Hu N., Fu S.-Y., Novoselov K.S. (2021). Graphene film for thermal management: A review. Nano Mater. Sci..

[29] Shuang F., Aifantis K.E. (2021). Dislocation-graphene interactions in Cu/graphene composites and the effect of boundary conditions: A molecular dynamics study. Carbon.

[30] Xie B., Li Q., Zeng K., Sahmani S., Madyira D.M. (2020). Instability analysis of silicon cylindrical nanoshells under axial compressive load using molecular dynamics simulations. Microsyst. Technol..

[31] Tian W., Li W., Liu X., Wang Y. (2017). Molecular dynamics study on the resonance properties of a nano resonator based on a graphene sheet with two types of vacancy defects. Appl. Sci..

[32] Han T., He P., Wang J., Wu A. (2010). The effect of vacancy defects on the tensile mechanical properties of single graphene sheets. J. Tongji Univ. Nat. Sci..

[33] Zandiatashbar A., Lee G.-H., An S.J., Lee S., Mathew N., Terrones M., Hayashi T., Picu C.R., Hone J., Koratkar N. (2014). Effect of defects on the intrinsic strength and stiffness of graphene. Nat. Commun..

[34] Wu Y.A., Fan Y., Speller S., Creeth G.L., Sadowski J.T., He K., Robertson A.W., Allen C.S., Warner J.H. (2012). Large single crystals of graphene on melted copper using chemical vapor deposition. ACS Nano.

